# Elemental Contamination in Indoor Floor Dust and Its Correlation with PAHs, Fungi, and Gram+/− Bacteria

**DOI:** 10.3390/ijerph16193552

**Published:** 2019-09-23

**Authors:** Sharif Arar, Afnan Al-Hunaiti, Mohanad H. Masad, Androniki Maragkidou, Darren Wraith, Tareq Hussein

**Affiliations:** 1 Department of Chemistry, School of Science, University of Jordan, Amman 11942, Jordan; a.alhunaiti@ju.edu.jo; 2 Water, Environment and Arid Region Research Center (WEARRC), Al al-Bayt University, Al-Mafraq 25113, Jordan; mohanad@aabu.edu.jo; 3 Finnish Meteorological Institute, Atmospheric Dispersion Modelling, P.O. Box 503, FI-00101 Helsinki, Finland; androniki.maragkidou@fmi.fi; 4 School of Public Health and Social Work, Queensland University of Technology, Queensland 4000, Australia; d.wraith@qut.edu.au; 5 Department of Physics, The University of Jordan, Amman 11942, Jordan; 6 Institute for Atmospheric and Earth System Research (INAR), University of Helsinki, PL 64, FI-00014 Helsinki UHEL, Finland

**Keywords:** ICP-OES, cluster analysis, Spearman correlation, dwellings, educational building

## Abstract

In this study, we performed elemental analysis for floor dust samples collected in Jordanian microenvironments (dwellings and educational building). We performed intercorrelation and cluster analysis between the elemental, polyaromatic hydrocarbon (PAH), and microorganism concentrations. In general, the educational building workshops had the highest elemental contamination. The age of the dwelling and its occupancy played a role on the elemental contamination level: older and more occupied dwellingshad greater contamination. The elemental contamination at a dwelling entrance was observed to be higher than in the living room. We found exceptionally high concentrations for Fe and Mn in the educational workshop and additionally, Hg, Cr, and Pb concentrations exceeded the limits set by the Canadian Council of Ministers of the Environment. According to the cluster analysis, we found three major groups based on location and contamination. According to the enrichment factor (*EF*) assessment, Al, Co, Mn, Ti, and Ba had *EF* < 2 (i.e., minimal enrichment) whereas P, S, Pb, Sb, Mo, Zn, Hg, and Cu had *EF* > 40 (i.e., extremely enriched). In contrast, Ca and P were geogenically enriched. Furthermore, significant Spearman correlations indicated nine subgroups of elemental contamination combined with PAHs and microbes.

## 1. Introduction

Pollution has been a general and common problem for a long time, affecting both indoor and outdoor environments [1]. Indoor pollution has been a focus of research for many years as people spend most of their time (more than 80%) indoors (e.g., dwellings, workplaces, offices, schools, etc.) [2,3,4,5]. Indoor floor dust has been reported in many studies as it acts a sink for various airborne pollutants including chemical pollution (e.g., heavy metals, polychlorinated biphenyls (PCBs), polyaromatic hydrocarbons (PAHs), and a vast range of organic compounds) and biological contamination (e.g., microorganisms, fungi, bacteria, viruses, insects and their dry parts, dustmites, and cells from humans, plants, and animals). These mixtures of pollutants can come from common or different sources but actually metabolize and act synchronically with each other in vivo and in vitro [6]. Indoor airborne pollutants might originate from either indoor or outdoor sources. In general, indoor sources of air pollution are closely linked to occupants’ activities and the use of household electronic devices, chemical products, building materials, and combustion processes (solid, liquid, and biomass fuels, etc.) [7,8,9,10,11,12,13,14,15]. Regardless of their source origin, they settle and accumulate in floor dust indoors.

Heavy metals are non-degradable, toxic, mutagenicity, and carcinogenicity. As a common type of indoor floor dust pollution, they have adverse health effects such as damage to the nervous system, cardiovascular deaths, slow growth development, and asthma [9,16,17,18,19]. Exposure to heavy metals can occur via three main pathways: dermal, inhalation, and ingestion [20,21]. Hand to mouth ingestion is a major pathway for small children and crawling infants. Therefore, assessment of heavy metal contamination in indoor floor dust is an important topic. However, risk assessment studies focused on a single type of pollution (e.g., organic, inorganic, or biological) and few studies presented combine investigations for heavy metals with other types of pollution and their sources and health risk assessments at the same time [22]. For instance, heavy metals in indoor floor dusts are considered an important indicator of urban microenvironments because they are linked to urban activities (e.g., tailpipe and none-tailpipe emissions as well as industrial and agricultural activities) and inhabitants activities besides degradation and use of furniture, appliances, building materials [13,23,24,25,26,27,28,29,30,31,32,33]. While many reports and studies about trace elements and heavy metals in indoor floor dust (e.g., educational buildings and residential areas) can be found in the literature [28,34,35,36], there are few reports in Jordanian microenvironments. For example, Al-Momani and Shatnawi [37] reported heavy metals concentrations in selected household dust in Jordan. Jardat et al. [38] reported the inorganic constituents of floor dust inside offices in an industrial area in Jordan. Al-Mdanatet al. [39] reported concentrations of heavy metals in indoor dust in Karak city, Jordan. Some of these studies were focused on the determination of a heavy metals content, health risk assessment, source origin, and spatial distribution employing multivariate analysis, enrichment factors, and contamination degree [40,41]. Organic pollutants may originate from open fires, unsafe combustion of biomass fuels including coal, charcoal and kerosene, tobacco smoking, gas stoves, and wood burning units. They are major sources of carbon monoxide and polyromantic hydrocarbons (PAHs). In addition, solid waste management and chemical spills could produce polychlorinated dibenzo-dioxins/furans (PCDDS/Fs) and polychlorinated biphenyls (PCBs). In addition to residual pesticides, such as pyrethroides and diethyltoluamide from spraying farms, homes, and schools. Volatile organic compounds (VOCs) are released from different sources including paints, glues, resins, polishing material, cleaning agents and household products. Microorganisms belong to biological pollutants that include animal dander, dust mites (fungi and bacteria), molds (Penicillium/Aspergillus), infectious and allergic agents (cat dander), and pollen. These organisms originate from indoor or outdoor environments. They proliferate in dirt, water damaged surfaces, air conditions, humidifiers, carpets, and furniture [6,7,11]. Recently, we also performed simple statistical correlations between the microbes and PAHs in selected indoor environments in Amman, Jordan [42].

In this study, we extended the analysis to quantify elemental contamination in indoor floor dust collected inside selected microenvironments (dwellings and an educational building) in Amman, Jordan. Dwellings and an educational building were studied due to the expected high concentrations of particulate matter, bio-aerosols, chemical pollutants based on area expansion and anthropogenic activities. We also investigated the relationship between heavy metals, microbes, and PAHs based on multivariate and cluster analysis. Understanding the correlations among pollutants could be an initial step for risk assessment of combined exposures to multiple associated chemicals of indoor floor dust contamination. According to the best of our knowledge, such analysis has not been done elsewhere.

## 2. Materials and Methods 

### 2.1. Floor Dust Samples

Floor dust samples were collected during 3–9 April 2015 from eight dwellings and an education building located in Amman, Jordan (Supplementary Material, Figures S1 and S2, and Tables S1 and S2). All these indoor microenvironments were naturally ventilated. The weather conditions during the sampling period are presented in Figure S3.

Two samples were taken from each dwelling (living room and main entrance). To collect a reasonable amount of floor dust samples, the dwellings were not vacuum cleaned for 3–4 days before sample collection. The educational building was the Department of Physics (University of Jordan), where all samples were collected on the same day. Although smoking was prohibited inside the university buildings, sometimes this was violated.

As described by Maragkidou et al. [43,44]. The floor dust samples were collected by using a regular vacuum cleaner equipped with vacuum dust bags (Allied Filter Fabrics Pty., Ltd., Berkeley Vale, Australia). The sample collection was for 3 min of vacuum cleaning. Immediately after dust collection, the dust bags were closed and put inside a zipped plastic bag. Each dust sample was then put in a glass vial, wrapped with aluminum foil and stored in the freezer (−20 °C) until the chemical and biological analysis was due.

### 2.2. Chemical and Biological Analysis

#### 2.2.1. Elemental Analysis

The elemental analysis was performed by applying inductively coupled plasma optical emission spectroscopy (ICP-OES). We analyzed nineteen elements: P, S, Al, Fe, Pb, Ca, Ba, Cr, Sb, Mo, Ti, Zn, Cd, Hg, Ni, Mn, Cu. Co, and Sr.

The dust samples were first digested before applying the elemental analysis. The used glassware were soaked overnight with 10% nitic acid then rinsed with distilled water followed by ultra-high-quality deionized water. Floor dust samples were dried overnight in an oven at 105 °C. We transferred 0.2–0.25 g of each dust sample (weighed to the nearest fifth digest with a Mettler Toledo 5-digit analytical balance AB 135S) into a glass (Duran) beaker for aqua regia digestion in an open system [45]. The digestion was made with 8 ml of 65% nitric acid (HNO_3_, analar, Fluka, Switzerland) and 3 mL of 35%–37% hydrochloric acid (HCl analar, Fluka, Reinach, Switzerland). The digested sampled in beakers were covered with watch glasses and heated to boiling (120–160 °C) for four hours. The watch glasses were then removed to allow solvent evaporation until the samples were dry. Then another 3 mL of 65% nitric acid was added and left to evaporate before reaching digested sample volume of about 0.5 mL. The solutions were diluted to 5 mL with ultra-high-quality deionized water (conductivity around 0.05501 µS/cm), then filtered with What man filter paper no 42 (0.45 μm) into 25 mL volumetric flask and tipped to the mark with ultra-high quality deionized water (Milli-Q purification system).

Elements determination with the ICP-OES was carried out in triplicate with 10% of the samples prepared in duplicate. A PerkinElmer instrument model optima 2000 DV was used operating with a Meinhard type c nebulizer and quartz torch. The following operating conditions were used: viewing height of 15 mm, replicate read time of 20 s per replicate, plasma gas flow of 15 L/min, auxiliary gas flow of 0.2 mL/min, sample aspiration rate of 0.8 mL/min, and pump flow rate of 1.5 L/min. The detection wavelengths for each element are listed in Table S3 and the calibration procedure and lower detection limit [46] are described in the supplementary material (see also Table S4). All measured elements showed high linearity with coefficients of determination (R^2^) ranging from 0.9706 for Al to 0.9997 for Sr. The method detection limits ranged from 0.004 µg/g for Sr to 122.88 µg/g for S as indicated in detail in Table S4. Recoveries ranged from 86.4% to 102% with an average recovery of 93%.

#### 2.2.2. PAHs and DNA Analysis

For the purpose of cluster analysis with other pollutants in the same samples, we recall the chemical and biological analysis of the same samples that was previously presented by Maragkidou et al. [43,44] and Al-Hunaiti et al. [42]. As mentioned, the main purpose of this manuscript was to focus on the heavy metals analysis and apply cluster analysis to group the chemical and biological contaminations against the indoor environments. As such, the PAHs and qPCR results (Tables S5–S8), which were previously discussed before by Maragkidou et al. [44] and Al-Hunaiti et al. [42], are not the main focus of this manuscript.

As described by Maragkidou et al. [44], the previous chemical analysis consisted of polycyclic aromatic compounds (PAHs), which are described in the supplementary material (see also Tables S5 and S6); and was performed by applying gas chromatography mass spectrometry (GC-MS). As described by Al-Hunaiti et al. [42], the biological analysis was performed by applying quantitative PCR (qPCR) analyses and it included total fungal DNA, group of *Aspergillus* spp./*Penicillium*, fungi, and Grampositive and Gramnegative bacteria.

### 2.3. Enrichment Factor (EF)

We sorted the elements sources as natural or anthropogenic (non-crustal)by using the enrichment factor (*EF*) Equation (1):

(1) $$EF=\frac{{\left(C_{x}/C_{ref}\right)}_{dust}}{{\left(C_{x}/C_{ref}\right)}_{crust}},$$

where *C_x_* is the concentration of the measured element and *C_ref_* is the concentration of the reference elements (here chosen to be Fe). The subscript *dust* refers todust samples whereas *crust* to crustal concentrations. Here five *EF* ranges can be assumed [47]: *EF* less than 2 as minimal enrichment, *EF* in the range 2–5 as moderate, *EF* in the range 5–20 as significant, *EF* in the range 20–40 as high, and *EF* more than 40 as extremely high.

### 2.4. Cluster Analysis

Hierarchical clustering was applied by running Cluster 3.0 program and using the average linkage method and Pearson correlation (centered correlation) as a similarity measure in order to decide on the appropriate number of clusters for our work as well as to investigate the association among PAHs, heavy metals and bio-contaminants inside the Jordanian dwellings and the university areas.

This program combines, arranges, and analyzes to show relationships between data elements. During the hierarchical analysis, all the data (genes and arrays) were mean centered in log-space and normalized. The visualization of clustered data was performed by tree diagrams-dendrograms generated with Java Tree View 1.1.6r4. Vertical analysis was based on dissimilarities among areas and groups. The horizontal analysis was based on ranking the indoor environments in similar groups based on contamination levels. In this analysis, all variables (different types of contaminants) are considered jointly (rather than separately as in Spearman’s correlation).

Spearman’s correlation was also estimatedfor correlated pollutants regardless to the indoor environments, where the following codes were used: PA (Penicillium/Aspergillus), G+ (gram positive bacteria), G-(gramnegative bacteria), F(fungi), PAH1(phenanthrene), PAH2 (anthracene), PAH3 (fluoranthene), PAH4 (pyrene), PAH5 (Benzo[a]anthracene), PAH6 (chrysene), PAH7 (benzo[b]fluoranthene), PAH8 (benzo[k]fluoranthene), PAH9 (benzo[j]fluornathene), PAH10 (benzo[a]pyrene), PAH11 (indeno[1,2,3-CD]pyrene), PAH12 (dibenz[a,h]anthracene), PAH13 (benzo[g,h.i]perylene).

## 3. Results and Discussion

### 3.1. Elemental Concentrations and Enrichment Factor

**Figure 1:**
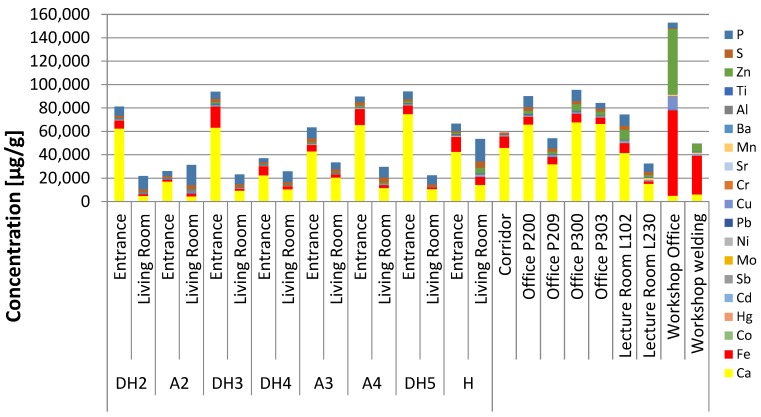
Elemental concentrations in the indoor floor dust.

The elemental concentrations of floor dust samples (described in detail in Figure S1 and S2, Tables S1 and S2) are listed in Table S9 and shown in Figure 1. According to the mean value and regardless to the sample collection location, the highest elemental concentration was found for Ca (28,500 µg/g, range 700–67, 800 µg/g), P (9200 µg/g, as high as 19,200 µg/g), Fe (8400 µg/g, range 900–72,900 µg/g), Zn (4500 µg/g; as high as 8500 µg/g), S (2800 µg/g, range 700–7900 µg/g), Al (700 µg/g, range 30–5500 µg/g), and Cu (500 µg/g, as high as 11,500 µg/g). The mean concentration was in the range 100–500 µg/g for Ba, Ti, Mn, and Sr whereas for Cr, Pb, Ni, and Hg it was between 10–100 µg/g. The rest of the elements (Mo, Sb, Cd, and Co) had mean concentrations less than 10 µg/g. The percent relative standard deviations (RSDs) for all samples elemental replicate analysis were ˂20%, which is the acceptable limit for digestion methods for trace analysis [48].

Inside the educational building (Figure 1, Table S10), the highest contaminated areaswere the WSA (workshop main room) and Wsb (metals cutting and welding area). The lecture room in the ground floor was higher in elemental contamination compared to offices on the first and second floors. The BC (main hall) located in the ground floor was the least contaminated. The results of total elemental concentration for the indoor environments were contrary or inversely proportional in general to the PAHs concentration results to the same environments reported previously by Maragkidou et al. [43,44].

The educational workshop area exhibited the highest concentrations of Fe (~72,900 µg/g and ~33,100 µg/g in two locations). This was mainly due to machinery and welding activities inside the workshop area. The Fe concentration reported here in this study exceeded what was previously reported by Jaradat et al. [38] in an industrial area (13,300 µg/g). The workshop area also had the highest total combined content of Zn (6400 µg/g), Cu (11,500 µg/g), and Mn (1300 µg/g) compared to other investigated areas in this study; these are also attributed to welding activities [29,30,49]. These concentrations exceeded what was previously reported in industrial offices [13,16,29,37,38]. Welding and machinery activities in the workshop area also lead to the highest concentrations of Pb, Cr and Hg among all samples. These concentrations were about 340 (Pb), 260 (Cr), and 80 (Hg) µg/g for Pb, Cr, and Hg, respectively, which exceeds the reported values in indoor dust reported in previous studies [13,16,29,37,38,49]. Here, the concentrations of Pb, Cr, and Hg exceeded the Canadian Council of Ministers of the Environment (CCME) limits [38,50]. The concentration of Hg was also high (about 130 µg/g) in a lecture room opposite to the first-year educational laboratories, where they use mercury in an educational experiment.

All dwellings’ floor dust samples showed varying concentrations of Pb, and Cr. Dwellings samples also indicated the presence of P and S elements in appreciable concentrations where the average ratio of P:S was 4:1 indicating crustal sources enriched with anthropogenic sources such as high temperature process (e.g., metal smelting, oil combustion and vehicular emissions) [51]. The comparison of elemental concentrations inside dwellings (entrance vs. living room) are demonstrated in Figure 1 (see also Table S10). In general, elemental concentrations at the entrance were higher than that was in the living rooms for all dwellings except for dwellings H and A2. For dwellings DH2, DH3, A3, A4, and DH5, the total elemental concentration ratio between living room and entrance area was 0.9, 0.5, 0.6, 0.8, and 0.6; respectively. As for A2 and H, ratio was 3.1 and 1.7; respectively. This ratio provides an indication about source origin (as indoor or outdoor) for the elemental contamination. Non-crustal elements with *EF* < 2 are relocated by dust fine particles as reported by Al-Momani and Shatnawi [37]; this confirms that contamination in living room for dwellings A2 and H was an indoor activity.

**Figure 2:**
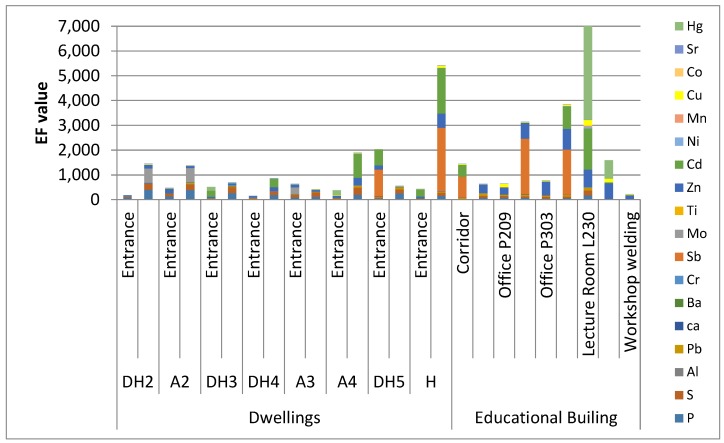
Enrichment factor for each indoor environment.

The *EF* values are shown in Figure 2 (see also Table S11). The elements Ba, Ti, Co, and Al had minimal enrichment *EF* (mean value <2) whereas Sr and Mn had moderate (mean value in the range 2–5). Elements (Al, Ti, Ba) coming from Khamaseen dust conssit partially of aluminum silicate minerals, and quartz; these elements reflect the natural lithological and mineralogical composition of the dust related material and source area [52]. Calcium is widely distributed in other minerals such as feldspar, amphibole and pyroxene, and is often associated with clay minerals such as illite, chlorite and Ca-montmorillonite. Ca is geogenically (naturally) enriched element as calcite which dominates the limestone deposition in the North African and Eastern Mediterranean dust (*EF* = 10) [52]. In general, the entrance area (*EF* in the range 5–14) of dwellings showed Ca concentrations higher than that found in the living rooms area. The corridor, lecture rooms, and offices in the educational building also showed high Ca concentration (*EF* in the range 7–16). *EF* values up to 10 are considered coming from the geogenic enrichment and more than 10 involved contribution from anthropogenic sources like cement factories, fertilizers especially that there was a construction site beside the educational building [52]. As for Cr, which is a toxic element with many adverse effects to humans [19,20]; it showed higher concentrations (*EF* = 10–28) in the living rooms than that in the entrances of the dwellings. This element is typically a by-product of fossil fuel combustion inside dwelling and from wood preservatives, chrome pigments (e.g., lead chromate) used in paints, printing inks, and anti-corrosive materials [26,27].

Mean *EF* > 40 (i.e., extremely high) was obtained for Hg, Sb, Cd, Zn, S, P, Mo, and Pb with mean values of *EF* = 1190, 364, 284, 248, 156, 145, 82, and 51; respectively. High *EF* indicates the presence of anthropogenic sources such as burning fossil fuels, power plants, metallurgy industry, maintenance equipment, workshops, chemical factories, pains and pigments [13,14,26] whereas *EF* < 5 indicates Saharan dust, which contains minor amounts of phosphorite [52].

### 3.2. Cluster Analysis and Intercorrelation of Pollutants

**Figure 3:**
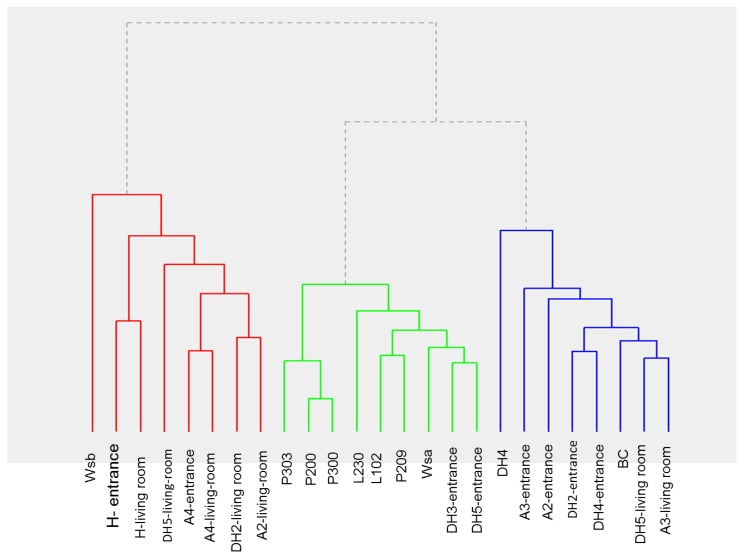
Hierarchical clustering (dendrogram) showing three major clusters.

We examined Spearman’s correlation coefficients at insignificant estimates with p-value threshold of 0.10 among three types of pollutants concentrations: elemental, biological (fungi and bacteria), and PAHs. According to the cluster analysis, we identified three groups based onmicro environment and total contamination (Figure 3). The first group (Group 1) included the workshop area at the educational building, entrance areas of dwellings H and A4, and living rooms areas of dwellings H, A4, A2, and DH2. The second group (Group 2) included the educational building premises and the entrance areas of dwellings DH3 and DH5. The third group(Group 3) included the entrance areas of dwellings DH2, A2, DH4, and A3 as well as the living room areas of dwellings DH3, DH4, and A3.

**Figure 4:**
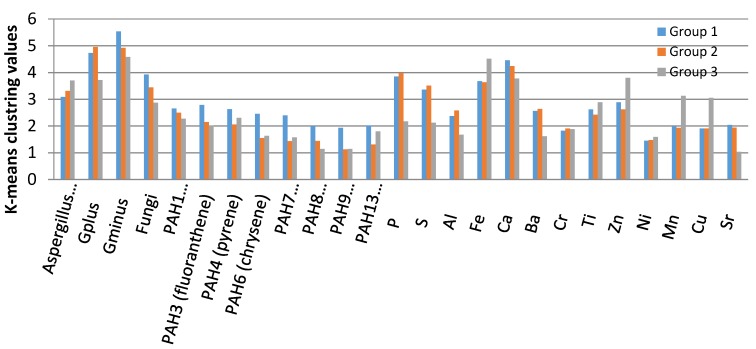
K-means clustering values of the three clusters with the different pollutants.

As shown in Figure 4, bacteria (gram positive and negative), *Penicillinum/Aspergillus*, and fungi are strongly associated with P, S, Fe, and Ca and to less extent with PAH1 (phenanthrene) for Groups 1 and 2 as demonstrated by the K-means clustering values. The major source of sulfurin the ambient environment is the combustion of sulfur-containing fossil fuels such as coal and crude petroleum. In addition, P and S are extremely enriched with values exceeding the 100 indicating the involvement of anthropogenic activity (e.g., combustion and vehicular emissions and fine particulate matter [51]) related to the area or locations in Group 1 and Group 2. As for Group 3, it showed bacteria (gram positive and negative), *Penicillinum/Aspergillus*, and fungi group strongly with Fe, Ca, Ti, Zn, Mn, Cu, and to a lower extent PAH4 (pyrene), PAH3 (fluoranthene), and PAH1 (phenanthrene) based on their K-means clustering values. The presence of extremely enriched Cu and high concentrations of Fe is a strong indication for anthropogenic activities. The low K-means clustering valuesof P and S in Group 3 compared to Groups 1 and 2 could be due to different sources and role of biological contamination [43,51].

**Figure 5:**
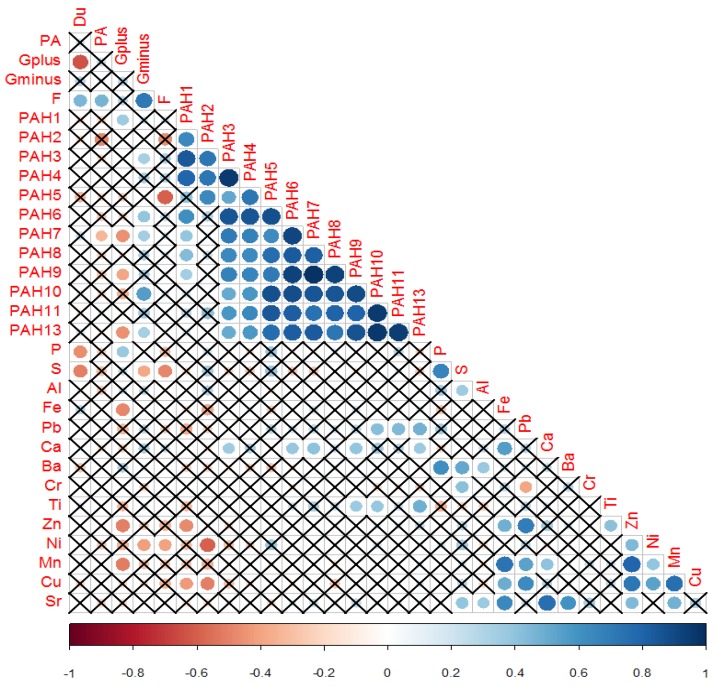
Plot of the correlation (Spearman) estimates for the variables analyzed (the size of the circles correspond to the absolute size of the correlation coefficient). Crosses refer to insignificant estimates at a *p*-value threshold of 0.10. Brown circles indicate negative correlation, blue circlesindicate positive correlation and insignificant correlation is marked as (×). The intensity of color is an indication for the value of the correlation coefficient (x-axis scale).

**Figure 6:**
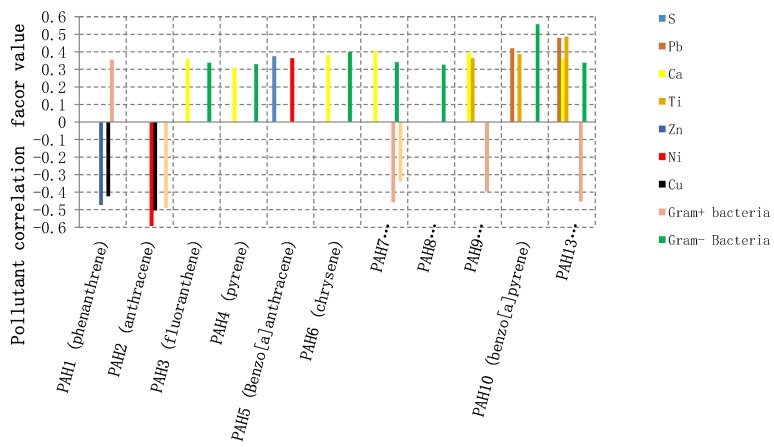
Bar presentation of the obtained correlation data of PAHs with gram positive bacteria, gramnegative bacteria, Penicillinum/Aspergillus, fungi, and elements.

The Spearman correlations are presented in Figure 5 (see also Figure 6). PAH1 (phenanthrene), which is from fossil burning and natural gas cooking, is positively correlated with gram positive bacteria (unable to metabolize the phenanthrene angular structure [42]) and negatively with the extremely enriched Cu and Zn, which could be coming from different anthropogenic sources (e.g., paints, cosmetics [27,28,29,43]). Gram positive bacteria correlates positively with P (correlation factor ~0.38 and *p* = 0.09), which could be explained may be by the presence of some gram positive bacterium strains that are reported to be responsible for phosphate solubilization and uptake like Bacillus spp. and Actino bacteria respectively, and are considered to be strong competitors in relation to other organisms [53,54]. Gram positive bacteria is negatively with Fe (correlation factor −0.48 and *p* = 0.02) and Zn (correlation factor −0.50 and *p* = 0.02), where these elements are consumed for essential and normal functioning of living organisms, extracellular and intracellular functions in addition to bio-sorption ability. Most microorganisms have surface antigens such as proteins, polysaccharides, teichoic acids, O-chain lipid oligo, and polysaccharides that have strong affinity and selectivity to bind or complex with a certain metal cation [55,56]. In addition, almost all species of bacteria originated from metal-rich environments, where bacteria uses metal cofactors to facilitate key cellular processes, such as production of energy and replication [57]. However, these elements could become toxic and lethal for microorganisms at high concentrations because of forming radical compounds in the cell of the microorganism [55,56,57], as noticed from the negative correlation.

PAH2 (Anthracene) showed negative correlations with Ni and Cu. This could be due to the fact that both Ni (significantly enriched) and Cu (extremely and highly enriched) come from different anthropogenic sources, such as tires, oil and gasoline additives, and metal parts in addition to certain types of pigments whereas anthracene could be coming from wood preservatives and diluents for pigments and colors [27,29,32,43]. This correlation could also indicate that *Penicillinum/Aspergillus* and other strains of fungi show high tolerance for toxic heavy metals, such as Ni and Cu [57,58].

PAH3 (fluoranthene), PAH4 (pyrene), and PAH6 (chrysene) are positively associated with gramnegative bacteria and Ca, while PAH7 (benzo[b]fluoranthene) is positively correlated with Gram negative bacteria and Ca and negatively associated with Gram positive bacteria and *Penicillinum/Aspergillus*. G+ bacteria is able to metabolite these PAHs in contrast to G- bacteria. The presence of strong association with Ca. PAH9 (benzo[j]fluornathene) was moderately associated with Ca, and Ti. PAH10 (benzo[a] pyrene) had a moderate positive association with gramnegative bacteria, Pb, and Ti. PAH11 (Indeno[1,2,3-CD]pyrene) was associated with Pb, which could be related to common anthropogenic sources, such as heating with kerosene, charcoal, and oil, in addition to using petroleum chemicals [43]. PAH13 (benzo[g,h.i]perylene) was associated with Pb, Ca, and Ti and negatively correlated with gram positive bacteria. In general, many factors influence the association between microorganism strain type and its ability to metabolite certain structure of PAHs depending on its active structural regions (bay region, bay-like region, M-region, and K-region) that end with Diol-epoxide active metabolic intermediates. In addition, the associationis dependent on the heavy metal anthropogenic common source and the microorganism tolerance or consumption for these heavy metals or elements [56,59,60]. Furthermore, the major metal-metal associations and minor PAH–metal or metal–bioaerosol associations indicates that the three classes of pollutants correlating in Figure 5 exhibit in general different emission resources, accumulation and metabolism pathways.

As also shown in Figure 5, Cu correlated with Zn (correlation coefficient 0.73 and *p* < 0.001), Ni (correlation factor 0.55 and *p* = 0.01), and Mn (correlation factor 0.75 and *p* < 0.001). In general, Cu and Zn are grouped as extremely enriched elements that can be related to wear parts of alloys, where Cu is used in different machinery parts and in brass automotive radiators whereas Zn is used in different parts of alloys and vehicles and as antioxidant and lubricant in oils. Also, Pb is associated positively with Zn and Cu; this indicates that these may be coming from the same sources [29,31,41].

Other metal–metal associations are indicated in Figure 5, where the significant strong correlations of metals are supported by an earlier section (Section 3.1) by enrichment factor grouping levels. In addition, we used skewness values for pollutants which provide information about the symmetric distribution of pollutants including metals, where the degree of deviation from symmetric distribution involves the contribution of anthropogenic activities [24,32,41,49] and supports data from enrichment factors and Spearman correlation data and grouping of pollutants explained in the text. Where skewness value less than −1 and greater than 1 is considered highly skewed, while values (−1 to −0.5 and 1 to 0.5) are considered to be moderately skewed, and values (−0.5 to 0.5) is considered symmetrically distributed (no anthropogenic involvement). After Investigation the relationship between pollutants regardless of the indoor environment for example: PA and F had skew values 3.6 and 4 (correlation 0.467), whereas PAH1, PAH3, and PAH4 have skew values 3.8,4.2, and 4.3 respectively which make them act as one group as in Figure 5 coming from the same source (correlation factors higher than 0.79. The same is applied for PAH11 and PAH13 with skew values 1.2, and 1.3 respectively and correlation coefficient of 0.9344. Zn and Cu skew values where 1.4 and 1.6 respectively (correlation 0.727) as in Figure 5 and for Mn and Cu 2.6 and 1.6 respectively (correlation 0.75). For example (PAH1, G+, Zn, Cu) they had skew values 4, 2.2, 1.4, and 1.6 respectively indicating high involvement of anthropogenic activity where average EF values were 248 and 31 respectively (significantly and extremely enriched) the four components had correlation around 0.4. These skew values for some extent were supportive for the data explanation mentioned above.

In general, we concluded from Pearson correlation factors for individual pollutants and skew values that the higher skew value, the higher enrichment factor for an element. In addition, the more the matching of skew values among different pollutants the more there will be the correlation to make the same group.

So in addition to PAH–PAH correlations, each PAH is correlated with metals and microorganisms to form its unique group (PAH–element–microorganism) as described in Figure 6 as in groups regardless if the correlation is positive or negative: (PAH1,G+,Zn, Cu), (PAH2, PA,Ni,Cu), (PAH3,Ca,G-), (PAH6,G-,Ca), (PAH7,G+,G-,PA,Ca), (PAH9,G+,Ca,Ti),(PAH10,G-,Ti,Pb), (PAH11,Pb), (PAH13,G+,G-,Ti, Pb,Ca).

## 4. Conclusions

In this study, we quantify elemental contaminations found in indoor floor dust samples that were collected inside some microenvironments (dwellings and an educational building) in Amman, Jordan. We also investigated the relationship between heavy metals, microbes, and PAHs based on multivariate and cluster analysis leading to the following major findings.

The average concentration of elements in µg/g in samples varied form extremely high amounts of Ca (~29,000 µg/g), and Fe (8400 µg/g) to moderate amounts of Ni (40 µg/g), and Cr (~90 µg/g), and lower concentrations of Cd (~5 µg/g) and Co (~2 µg/g). In general, the educational building workshops (~49,400 µg/g) were the highest contaminated by elements followed by the living room for H dwelling (66,500 µg/g), While the lowest concentration in indoor environments was the main entrance area for A2 dwelling (2nd floor apartment) located in the north eastern part of the capital Amman. In addition, for dwellings, the elemental contamination in the entrance was found to be greater than the living room for all dwellings except for dwelling H (entrance < living room) and dwelling A2 (entrance < living room).

Pollution assessment by mean values of EF of these elements indicated which elements are extremely enriched and which elements are with minimum enrichment as: Hg (~1110), Sb (~350), Cd (~300), Zn (~250), S (~160), P (~150), Mo (~80), Pb (~50), Cu (~30), Cr (~15), Ni (~7), Ca (~7), Sr (~4), Mn (~2), Ba (~1), Ti (~1), Co (~1), Al (~ 0).Where Al, Co, Mn, Ti, and Ba are considered with minimal enrichment <2.Sr is considered moderate enrichment whereas Ca, Ni, Cr are considered to be significantly enriched. The other elements P, S, Pb, Sb, Mo, Zn, Hg, and Cu are considered to be extremely highly enriched in general. In contrast, Ca, and P were geogenically enriched.

Investigation of the relationship between pollutants (regardless of the indoor environment) resulted in a potential grouping based on sources for pairs or a variety of pollutants which would be of high probability of common source of emissions including (PA and F), (phenanthrene, Anthracene, and fluoranthene), (Indeno[1,2,3-CD]pyrene and benzo[g,h.i]perylene), (Zn and Cu), and (Mn and Ni). This could be very useful in health risk assessment based on combined exposures to multiple associated pollutants. The Spearman correlation analysis was at 0.1 significance level and was confirmed by skew values for each pollutant indicated major significant correlations for PAH–PAH, metal–metal, microorganism–microorganism.

Furthermore, the important major finding in this study reveals a significant number of tri-component correlations related to PAH–metal–microorganism including:(phenanthrene, G+, Zn, Cu), (Anthracene, PA, Ni, Cu), (fluoranthene, Ca, G-), (chrysene, G-, Ca), (benzo[b]fluoranthene, G+, G-, PA, Ca), (benzo[j]fluornathene, G+, Ca, Ti), (benzo[a]pyrene, G-, Ti, Pb), (Indeno[1,2,3-CD]pyrene, Pb), (benzo[g,h.i]perylene, G+, G-, Ti, Pb, Ca). Many factors influence the association between microorganism strain type and its ability to metabolite certain structure of PAHs depending on its active structural regions (bay region, bay-like region, M-region, and K-region) that end with Diol-epoxide active metabolic intermediates. In addition, an association is dependent on heavy metal anthropogenic common source and the microorganism tolerance or consumption for these heavy metals or elements. Given the complexity of the data analysed, the major metal-metal associations and minor PAH–metal or metal–bioaerosol associations indicate that the three classes of pollutants exhibit in general different emission resources, accumulation and metabolism pathways.

## Supplementary Materials

The following are available online at https://www.mdpi.com/1660-4601/16/19/3552/s1, Figure S1: A map of Amman with site locations of the dwellings marked with yellow landmarks (abbreviations as listed in Table S1) and the campus of the University of Jordan (marked with red, see also Figure S2 and Table S2); Figure S2: A map of the University of Jordan campus and a schematic chart of the Department of Physics with indications for the rooms from where floor dust samples were collected; Table S1: Features of the dwellings and floor, where the dust samples were collected; Table S2: A summary about the rooms and floor surfaces from which the dust samples were collected at the Department of Physics, the University of Jordan. Samples were collected on April 29, 2015. The bare floor tiles used in the building are cement filled with small marble stones; Figure S3:Ambient temperature, relative humidity, and accumulated rain (since April 1st); Table S3: ICP-OES detection wavelengths (nm); Table S4: Calibration curve equation for measured elements; Table S5: Polycyclic aromatic hydrocarbons (PAHs [ng/g]) concentrations in the dust samples collected from the dwellings (Maragkidou et al., 2016); Table S6: Polycyclic aromatic hydrocarbons (PAHs [ng/g]) concentrations in the dust samples collected from the educational building (Maragkidou et al., 2017); Table S7: Microbe concentration [cell equivalent/mg] based on the qPCR-DNA analysis of the dust samples collected from the dwellings; Table S8: Microbe concentration [cell equivalent/mg] based on the qPCR-DNA analysis of the dust samples collected from the educational building; Table S9: Elemental concentrations [µg/g] of the floor dust samples; Table S10: Total measured elemental concentration; Table S11: Average enrichment factor (EF) and range for measured elemental concentrations.
