# Supplementary material for: Elemental Contamination in Indoor Floor Dust and Its Correlation with PAHs, Fungi, and Gram+/− Bacteria
Source: Int J Environ Res Public Health. 2019 Sep 23;16(19):3552. doi: 10.3390/ijerph16193552 (PMC6801931; doi:10.3390/ijerph16193552)
Supplement: Supplementary file 1 [file ijerph-16-03552-s001.pdf]

Supplementary Material

Elemental Contamination in Indoor Floor Dust and its Correlation with PAHs, Fungi, and +/-Gram Bacteria

Arar et al.

1. Site locations and sample collection

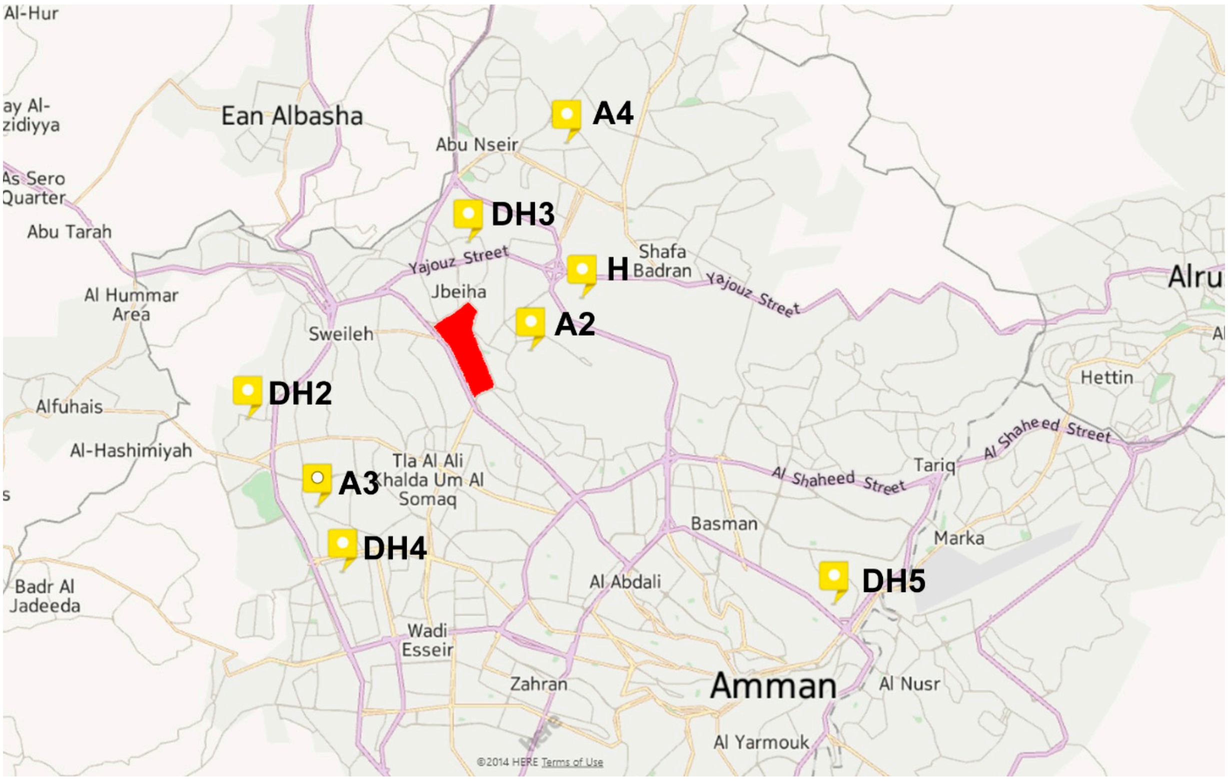

Figure S1. A map of Amman with site locations of the dwellings marked with yellow landmarks (abbreviations as listed in Table S1) and the campus of the University of Jordan (marked with red, see also Figure S2 and Table S2).

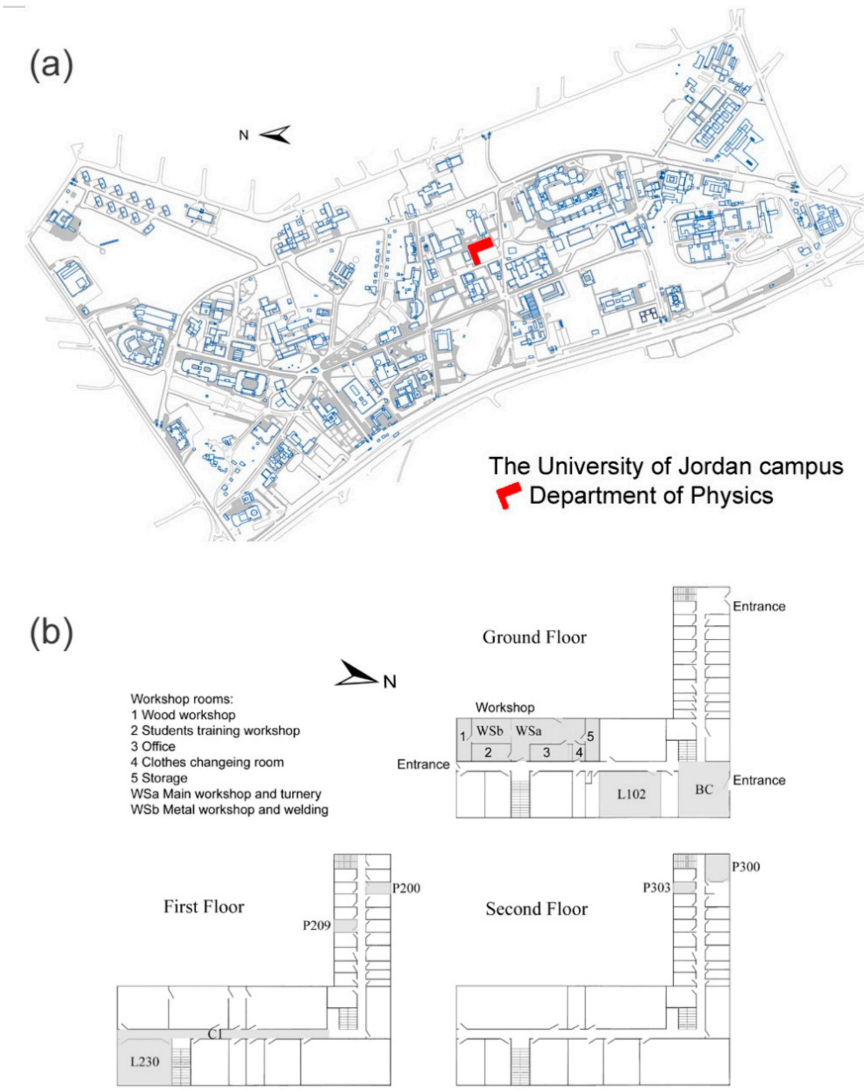

Figure S2. A map of the University of Jordan campus and a schematic chart of the Department of Physics with indications for the rooms from where floor dust samples were collected.

Table S1. Features of the dwellings and floor, where the dust samples were collected.

Code	Age [years]	Type	Level in Building	Location in Dwelling	Area [m^2], Type	Date
DH2	10	Detached House	2nd floor	Entrance	0.75 × 0.50, carpet	April 3
				Living room	1.00 × 1.00, carpet	
A2	5	Apartment	2nd floor	Entrance	0.91 × 0.60, carpet	April 3
				Living room	1.00 × 1.00, carpet	
DH3	30	Detached House	Ground floor	Entrance (inside)	2.00 × 2.00, carpet	April 3
				Living room	2.00 × 2.00, carpet	
DH4	15	Detached House	Ground floor	Entrance (inside)	0.60 × 0.60, carpet	April 5
				Living room	2.00 × 2.00, carpet	
A3	4	Apartment	1st floor	Entrance (inside)	1.00 × 0.50, carpet	April 5
				Living room	2.00 × 2.00, carpet	
A4	3	Apartment	2nd floor	Entrance (inside)	2.00 × 2.00, ceramic tiles	April 3
				Living room	2.00 × 2.00, carpet	
DH5	40	Detached House	Ground floor	Entrance (inside)	2.00 × 2.00, carpet	April 3
				Living room	2.00 × 2.00, carpet	
H	10	House	Ground floor	Entrance	1.00 × 1.00, ceramic tiles	April 9
				Living room	2.00 × 2.00, ceramic tiles	

Note: In this study, an "apartment" is a dwelling in an apartment building that shares the main entrance of the building and some common areas around the building (such as parking and the roof). A "house" is built on its own separate land. A "detached house" is an apartment in the ground floor of an apartment building but it has its own entrance and facilities; in that sense, it is like a house in living style but similar to apartments in structure.

Table S2. A summary of the rooms and floor surfaces from which the dust samples were collected at the Department of Physics, the University of Jordan. Samples were collected on April 29, 2015. The bare floor tiles used in the building are cement filled with small marble stones.

			**Surface area [m^2]**	**Surface type**
P200	Office 200	1st floor	1.50 × 1.50	Carpet
P209	Office 209	1st floor	3.00 × 3.00	Bare floor
P300	Office 300	2nd floor	3.00 × 1.00	Carpet
P303	Office 303	2nd floor	1.50 × 1.50	Carpet
L230	Lecture room 230	1st floor	2.70 × 1.35	Bare floor
L102	Lecture room 102	Ground floor	3.90 × 1.50	Bare floor
WSa	Workshop, main area	Ground floor	1.50 × 1.50	Bare floor
WSb	Workshop, welding area	Ground floor	1.50 × 1.50	Bare floor
C1	1st floor corridor	1st floor	1.80 × 3.00	Bare floor
BC	Big Corridor	Ground floor	2.70 × 2.70	Bare floor

2. Weather conditions

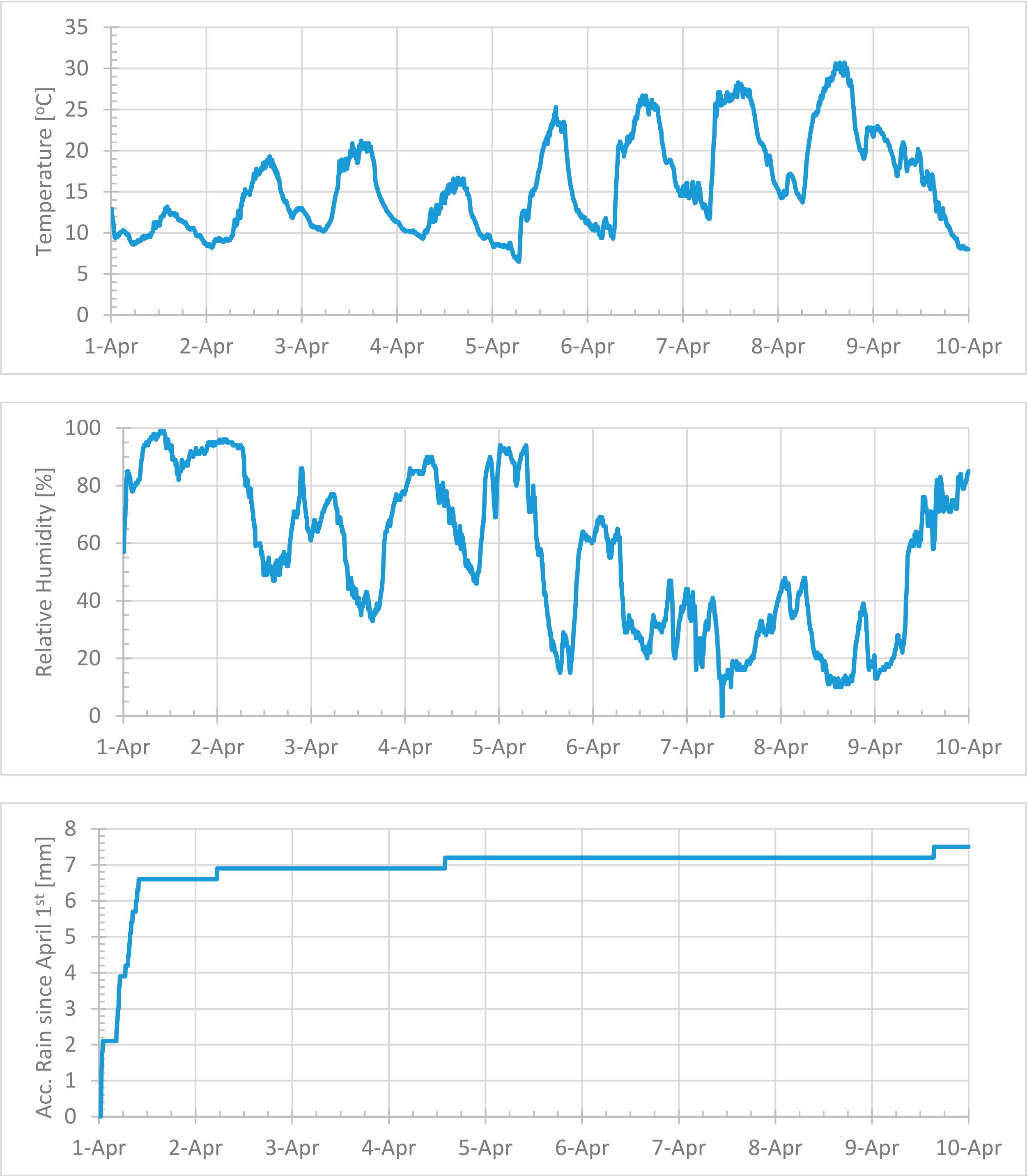

Figure S3. Ambient temperature, relative humidity, and accumulated rain (since April 1st).

3. Calibration and lower detection limit:

For calibration curve standards, the multi-element standard solution (PerkinElmer pure plus, PE# N9301721) was used and diluted with 2% nitric acid. In addition, individual standards of sulfur (N9303796), phosphorous (N9303788) and Mercury (N9300174) were diluted in the same way. A six-point calibration curve for each element was done (0.025, 0.05, 0.1, 0.5, 1.0, 2.0) mg/l. Reported concentrations were in μg/g and calculated based on Equation (1). Method blanks were prepared for method detection calculations; in addition, quality control certified reference material (Trace CERT-sigma-aldrich-90243-100ML) at three calibration levels low (0.05 mg/l), medium (0.1 mg/l) and high (0.5 mg/l) as check standards for accuracy and recovery calculations. Where the method blank and spiked samples were subjected to the digestion acid mixture. The method detection limit (MDL) for each element was calculated as below according to Equation (2):

$$\text{Conc. } [\mu g/g] = [(\text{determined conc. } [mg/L]) \times \text{volume } [mL] \times \text{dilution factor/weight of sample } [g] \quad (1)$$

$$\text{MDL} = t \times \text{ SD (at 99\% confidence level, where } t = 3.71 \text{ at six degrees of freedom, SD: standard deviation)} \quad (2)$$

All measured elements showed high linearity with coefficients of determination (R^2) ranging from 0.9706 for Al to 0.9997 for Sr as described in detail in Table S4. The method detection limits ranged from 0.004μg/g for Sr to 122.88 μg/g for S as indicated in detail in Table S6. Recoveries ranged from 86.4% to 102% with average recovery of 93%.

Table S3. ICP-OES detection wavelengths (nm).

**P**	**213.617**
S	181.975
Hg	253.652
As	193.696
Zn	206.200
Al	396.153
Cd	228.802
Ni	231.604
Mn	257.610
Cu	327.393
Sr	407.771
Co	228.616
Ti	334.940
Mo	202.031
Sb	206.836
Cr	267.716
Ba	233.527
Ca	317.933
Pb	220.353
Fe	238.204

Table S4. Calibration curve equation for measured elements.

Element	Calibration equation	Coefficient of determination (R^2)	Method detection limit (MDL)(μg/g)
P	Y= 3406x+211.48	0.9995	30.88
S	Y= 883.64x+19.036	0.9991	122.88
Al	Y= 145519x+8296.3	0.9706	7.46
Fe	Y=55875x + 905.89	0.998	1.9
Pb	Y= 5412x-130.24	0.9986	19.6
Ca	Y= 101458x-890.21	0.9981	10.7
Ba	Y= 150356x+2004.3	0.9996	7.22
Cr	Y= 71283x+282.83	0.9996	1.25
Sb	Y= 1701.7x+63.934	0.998	30.52
Mo	Y= 41659x+113.27	0.9964	20.01
Ti	Y= 1×10^6x-43676	0.9982	0.11
Zn	Y= 4456.3x+87.02	0.999	20.1
Cd	Y= 128455x-417.82	0.9994	1.3
Hg	Y= 12980x-359.86	0.9984	0.895
Ni	Y= 55400x-671.27	0.997	1.96
Mn	Y=2×10^6x-15593	0.9973	0.054
Cu	Y=83580x+5895.4	0.9933	0.85
Co	Y=14632x+590.72	0.9924	7.01
Sr	Y=3×10^7x-52653	0.9997	0.004

Table S5. Polycyclic aromatic hydrocarbons (PAHs [ng/g]) concentrations in the dust samples collected from the dwellings (Maragkidou et al., 2016).

Location	Dwelling	PHE	ANT	FLA	PYR	BaA	CHR	BbF	BkF	BjF	BaP	IcdP	DahA	BghiP
Entrance	DH2	498	--	2037	354	44	191	324	90	90	23	46	--	55
	A2	169	--	147	157	65	104	116	67	51	64	60	--	111
	DH3	241	--	206	185	117	184	261	104	101	97	71	20	118
	DH4	1016	100	3879	997	114	530	308	113	94	45	52	--	60
	A3	4973	806	2039	946	--	51	26	24	11	10	--	361	19
	A4	197	--	110	89	--	44	40	22	15	12	15	--	26
	DH5	275	--	261	211	59	110	72	42	25	33	31	--	59
	H	91	--	53	47	--	14	12	8	5	--	--	--	11
Living Room	DH2	355	--	138	122	--	128	139	69	42	--	39	--	49
	A2	161	--	65	67	--	37	36	144	--	--	--	172	27
	DH3	512	98	562	448	175	236	242	89	72	74	53	--	76
	DH4	14413	2397	26630	15780	2184	2377	621	286	219	181	152	--	181
	A3	1273	259	2454	1685	402	468	197	97	68	86	81	150	88
	A4	239	--	91	84	--	32	28	23	10	8	--	107	20
	DH5	462	--	153	118	--	48	30	37	12	10	--	37	24
	H	197	--	111	102	--	42	32	24	13	9	--	149	23

Table S6. Polycyclic aromatic hydrocarbons (PAHs [ng/g]) concentrations in the dust samples collected from the educational building (Maragkidou et al., 2017).

Location		PHE	ANT	FLA	PYR	BaA	CHR	BbF	BkF	BjF	BaP	IcdP	DahA	BghiP
Office	P200	380	168	810	672	487	763	603	258	241	222	241	--	307
Office	P209	438	105	393	275	153	199	167	78	65	98	60	--	94
Office	P300	410	106	969	723	467	739	693	241	220	253	174	--	252
Office	P303	220	84	490	439	283	534	500	183	167	182	152	--	210
Lecture room	L102	186	77	186	182	107	168	159	73	61	69	61	33	83
Lecture room	L230	316	99	264	243	141	199	212	86	68	101	83	--	129
Workshop, main area	WSa	171	--	251	147	--	147	270	32	81	--	55	--	93
Workshop, welding area	WSb	190	--	106	205	--	43	38	14	14	22	19	--	63
1st floor Corridor	C1	710	168	632	517	374	496	415	184	164	255	147	89	213
Big Corridor	BC	471	74	519	489	224	334	262	110	96	137	77	--	123

Table S7. Microbe concentration [cell equivalent/mg] based on the qPCR-DNA analysis of the dust samples collected from the dwellings.

Location	Dwelling	Gram-positive bacteria	Gram-negative bacteria	Penicillium Aspergillus spp.	Total fungal
Entrance	DH2	ND	1278700	600	14200
	A2	48200	1779800	7300	172300
	DH3	36700	246100	2600	10000
	DH4	29000	190500	1100	15300
	A3	348300	111300	1300	5700
	A4	17700	8900	200	500
	DH5	95800	478500	1900	16600
	H	ND	2254200	5500	35100
Living Room	DH2	NA	NA	NA	NA
	A2	49700	57200	1300	1400
	DH3	173500	254900	3900	30200
	DH4	11300	351200	1100	4400
	A3	959100	423000	2000	7700
	A4	77200	20700	600	600
	DH5	174500	82900	5400	3700
	H	265400	130900	26400	4200

Table S8. Microbe concentration [cell equivalent/mg] based on the qPCR-DNA analysis of the dust samples collected from the educational building.

Location		Gram-positive bacteria	Gram-negative bacteria	Penicillium Aspergillus spp.	Total fungal
Office	P200	46600	376000	600	3800
Office	P209	109300	315900	500	2700
Office	P300	20200	302600	600	3300
Office	P303	21300	288000	1500	7900
Lecture room	L102	44500	225600	1400	5100
Lecture room	L230	24700	195300	1100	7200
Workshop, main area	WSa	8300	89700	700	2000
Workshop, welding area	WSb	5300	38700	5100	700
1st floor Corridor	C1	136000	124400	200	2700
Big Corridor	BC	107400	495000	2300	10700

Table S9. Elemental concentrations [μg/g] of the floor dust samples.

Sample	P	S	Al	Fe	Pb	Ca	Ba	Cr	Sb	Mo	Ti	Zn	Cd	Hg	Ni	Mn	Cu	Co	Sr
DH2-entrance	7741	1950	545	6875	<MDL	6233	537	317	<MDL	<MDL	398	187	<MDL	0	19	100	25	<MDL	145
DH2-living room	11530	2459	156	1532	<MDL	4658	559	63	<MDL	20	477	266	<MDL	0	25	30	50	<MDL	52
A2-entrance	4357	1163	328	1732	<MDL	17101	359	34	<MDL	<MDL	501	364	<MDL	0	10	18	33	<MDL	70
A2-Living room	17320	3419	1851	2376	40	4448	840	80	<MDL	31	445	233	<MDL	0	38	40	30	<MDL	91
DH3-entrance	6113	2795	92	18024	30	63198	853	193	<MDL	<MDL	889	1238	13	4	20	186	86	<MDL	175
DH3-living room	8215	2573	272	1692	17	9319	377	110	<MDL	<MDL	342	136	<MDL	0	55	35	19	<MDL	54
DH4-entrance	3413	1598	56	7782	26	22348	335	68	<MDL	<MDL	467	572	<MDL	0	13	97	20	<MDL	173

DH4-living room	9227	2353	306	2640	<MDL	10365	60	45	<MDL	<MDL	197	496	3	0	29	58	6	<MDL	38
A3-entrance	5990	3417	313	5503	35	42899	537	97	<MDL	32	87	810	<MDL	0	22	69	93	<MDL	152
A3-living room	4639	2779	510	2621	17	20460	190	132	<MDL	<MDL	439	100	<MDL	0	13	36	40	<MDL	70
A4-entrnace	9060	2962	341	13565	22	65450	301	130	<MDL	<MDL	226	1255	<MDL	4	89	206	244	<MDL	229
A4-living room	6748	4475	293	2462	48	11572	261	69	<MDL	<MDL	245	869	7	<MDL	26	58	48	5	64
DH5-entrance	6748	1651	440	7485	50	74719	515	48	32	<MDL	451	1556	14	<MDL	16	175	53	<MDL	165
DH5-living room	7600	1623	263	1600	36	10668	179	38	<MDL	<MDL	265	3	<MDL	<MDL	14	41	15	<MDL	8
H-entrance	6525	1290	433	12684	<MDL	42519	533	59	<MDL	<MDL	876	1134	11	<MDL	21	238	78	<MDL	261
H-living room	19173	5503	252	6786	62	14254	1164	121	70	<MDL	363	4679	38	0	31	250	601	16	172
BC	<MDL	1791	249	9716	100	45904	441	48	33	<MDL	140	73	14	<MDL	<MDL	<MDL	299	7	144

P200	9142	3195	507	6569	143	65881	547	121	<MDL	<MDL	722	2747	<MDL	<MDL	24	106	108	<MDL	191
P209	8715	2836	321	6054	64	31898	408	6	<MDL	<MDL	551	2098	<MDL	<MDL	47	101	937	<MDL	109
P300	9460	2790	397	7311	125	67759	667	48	66	<MDL	555	5678	<MDL	<MDL	23	201	174	11	190
P303	4742	2343	68	5528	74	66414	208	59	<MDL	<MDL	652	3548	<MDL	<MDL	76	91	189	<MDL	137
L102	9556	3363	849	8406	130	41508	788	74	61	<MDL	204	8486	23	<MDL	45	221	433	12	209
L230	7033	2716	28	2215	61	15077	189	75	<MDL	<MDL	494	1885	11	125	219	1667	558	<MDL	56
Wsa	4608	705	30	72871	340	4998	272	261	<MDL	<MDL	199	55719	7	82	135	1092	11505	<MDL	41
Wsb	149	135	48	33084	107	6048	42	77	<MDL	0	785	6399	0	22	39	1343	1135	<MDL	11

Table S10. Total measured elemental concentration.

Sample (indoor environment code)	Total elemental concentration with EF>2 and excluding Ca
DH2-entrance	17114
DH2-living room	15944
A2-entrance	7693
A2-Living room	23566
DH3-entrance	28516
DH3-living room	12817
DH4-entrance	13492
DH4-living room	14798
A3-entrance	19282
A3-living room	11692
A4-entrnace	22910
A4-living room	17069
DH5-entrance	17654
DH5-living room	10929
H-entrance	21802
H-living room	37082
BC	12081
P200	22049
P209	20756
P300	25686
P303	16560
L102	30590
L230	14899
Wsa	146233
Wsb	41146

Table S11. Average enrichment factor (*EF*) and range for measured elemental concentrations.

Element	Average EF	Range
P	144.89	0-423.56
S	155.45	0.65-258.78
Al	0.137	0.0034-0.84
Pb	51.039	0-272.13
Ca	6.719	0.0926-13.552
Ba	1.446	0-4.486
Cr	15.14	0.52 -34.18
Sb	363.92	0- 3465.66
Mo	81.9	0- 576.33
Ti	1.15	0.0275- 3.145
Zn	248.14	0- 884.53
Cd	283.54	0- 1658.92
Hg	1189.63	0- 3796.46
Ni	7.37	0- 66.08
Mn	2.35	0-44.27

Cu	30.88	0-229.07
Co	1.07	0-5.93
Sr	4.03	0- 31.84
